# Serological and Molecular Evidence of Patients Infected with *Anaplasma phagocytophilum* in Mexico

**DOI:** 10.3390/diseases9020037

**Published:** 2021-05-14

**Authors:** Carolina Guadalupe Sosa-Gutierrez, Maria Almudena Cervantes-Castillo, Ramon Laguna-Gonzalez, Laura Yareli Lopez-Echeverria, Deyanira Ojeda-Ramírez, Mayra Oyervides

**Affiliations:** 1 Instituto de Ciencias Agropecuarias, Universidad Autónoma del Estado de Hidalgo, Tulancingo, Hidalgo 43600, Mexico; exilefenix93@gmail.com (R.L.-G.); emvzlaura@hotmail.com (L.Y.L.-E.); dojeda@uaeh.edu.mx (D.O.-R.); 2 BioGeneticks and Other Vector Diseases Lab., Tulancingo, Hidalgo 43660, Mexico; almu14@hotmail.com (M.A.C.-C.); moyervides@hotmail.com (M.O.); 3 Medicina Basada en Evidencia, Hospital Infantil de México “Federico Gómez”, Mexico City 06720, Mexico; 4 Departament of Biology, Schreiner University, Kerrville, TX 78028, USA

**Keywords:** *Anaplasma phagocytophilum*, zoonosis, humans, serology, molecular

## Abstract

Human granulocytic Anaplasmosis (HGA), is a tick-borne infectious disease transmitted by ticks, resulting in acute feverish episodes. The etiological agent is the bacteria *Anaplasma phagocytophilum*; which is spread by ticks of the genus *Ixodes spp*. to complete its life cycle. In Mexico, there is only one case report. The primary challenge is understanding how other bacteria affect or overlap with the clinical manifestation of the disease. Sample collection occurred over the period September 2017 through October 2019. Blood samples from human subjects were obtained immediately after they signed consent forms. We analyzed for the presence for *A. phagocytophilum* by serological (IFA IgG two times) and PCR targeting *16SrRNA* and *groEL* genes, followed by DNA sequencing. All patients with a history of travel abroad were dismissed for this project. In total, 1924 patients participated and of these, 1014 samples across the country were analyzed. Of these, 85 (8.38%) had IFA results that ranged from 1:384 to 1:896. Of the positive samples, 7.10% were used for PCR. Significant clinical manifestations included: dizziness, nausea, petechial, epistaxis, enlarged liver and/or spleen and thrombocytopenia. Hospitalization of at least 1.5 days was necessary for 3.2% of patients. None of the cases analyzed were lethal. This is the first clinical manifestations along with serological test results and molecular analysis confirmed the presence of *A. phagocytophilum* resulting in HGA in patients from Mexico. Health institutions and medical practitioners in general should include diagnostic testing for HGA among high risk populations and should recognize it as a vector-borne emerging infectious disease in Mexico.

## Impacts

There are patients with clinical suspected anaplasmosis in Mexico without diagnosis.

There is limited knowledge on the epidemiology and clinical characterization of patients, because of it is not a reportable disease in Mexico, and its presence is unknown.

Our discoveries will help to increase the clinical suspicion by clinicians, and therefore perform successful diagnosis for the improvement of patients. It is necessary to conduct a seroepidemiological study of the human relationship between host-vector-reservoir in Mexico.

## 1. Introduction

Human Granulocytic Anaplasmosis (HGA) is an infectious disease transmitted by ticks. Its manifestation results in acute feverish episodes and the etiological agent causing this is the bacterium *Anaplasma phagocytophilum*. *A phagocytophilum* completes its life-cycle through ticks of the genus *Ixodes spp*. and the principal reservoir for *A. phagocytophilum* is the white-footed mouse, *Peromyscus leucopus* [1]. Other mammals, such as “white-tailed deer” *Odocoileus virginianus,* may also carry the bacterium and serve as long-term asymptomatic hosts [2]. Other mammals occur, such as squirrels, voles, wood rats, roe deer, deer, cats, dogs, horses, ruminants and other sylvatic species that serve as reservoirs of diseases [3,4]. The distribution of this disease is related directly to the distribution of the vector. The infection has been reported globally [5].

*Anaplasma phagocytophilum* is an obligate intracellular and Gram negative bacterium, with a specific tropism towards leukocytes and platelets. These bacteria thrive and multiply within the cytoplasmic vacuoles of the host’s cells, thereby evading neutrophils and the antimicrobial functions of the host’s immune system. The infection is acquired through tick bites. Once in the host it disseminates to the bone marrow and the spleen, provoking within human patients a decrease in the elemental functions of these [2,4,5]. Symptoms are manifested within 5–14 days after the bite from an infected tick and generally the clinical manifestations vary from mild to severe and may include fever (92–100%), general discomfort (97%), myalgia (77%), headache (82%), and in less than 50% of cases vomiting, nausea, diarrhea and coughing. Effects to the central nervous system is rare [6]. Severe manifestation of the diseases may result in labored breathing, septic shock, multi-organ failure and rhabdomyolysis, as well as opportunistic infections [7,8]. However, there have been reports of peripheral neuropathy, thromboembolic pathologies, hemorrhagic manifestations, pancreatitis and acute renal failure [6].

Possible factors affecting the severity of Anaplasmosis manifestation may include being elderly, immunosuppressed, medical conditions such as diabetes and a delay in positive diagnosis and treatment of the disease. Fatality rates are greater for persons over 70 years of age and those with immunosuppression [6,8]. The majority of cases have been reported in the United States of America; nevertheless, reports containing serological evidence in Latin America, including Mexico, exist, particularly in a patient engaged in high risk activity [9]. A positive diagnosis may be confirmed through various methods, such as serological tests and examination of blood smears, which would reveal the presence of morulas within the granulocytes, DNA detection through PCR testing, bacterial detection through the examination of histological samples (such as those from bone marrow, spleen, lymphatic nodes, liver, or pulmonary tissues), or through culture isolation [4,6]. Detection through PCR testing of DNA is sensitive and specific for the diagnosis of acute anaplasmosis [9,10].

Tick-borne diseases are a serious public health concern, as the reported incidence of tickborne diseases has increased during the past decade and has erupted in areas not previously reported such as Ecuador, Chile and Mexico [11,12,13]. These have caused serious illness and death in children and adults, regardless of the availability of adequate treatment, such as early diagnosis [6]. Presently, Mexico only recognizes *Rickettsia rickettsii*, which has also been the cause of the latest outbreaks in the country since 2009, resulting in fatalities in 12% of those infected [13,14]. Throughout the northern part of the country, concerted efforts are being made to better understand the depth of this public safety problem [1,11]. There is a lack of information regarding how other bacteria or the overlap of other bacteria affect the clinical manifestations of the infection. The presence of the suitable vector as well as known reservoirs in the sylvatic life cycle of the bacterium has been reported in Mexico [9,11,15,16,17]. Coupled with the quantity of children, elderly adults and middle-aged adults presenting clinical symptoms (fever, headache, nausea, vomiting, abdominal discomfort, diarrhea, arthralgia, myalgia, hepato-splenomegaly, gingivohemorraging, petechial and increase transaminase levels, among some) [2]; are suggestive for hemorrhagic syndromes such as Zika, Chikungunya, classic and hemorrhagic Dengue, and of course tick-borne diseases. Hence, it is of interest to understand the frequency and clinical characterization of a series of suspect patients with HGA acquired in the Mexican republic, employing the international legislative criteria for diagnosis, since Mexico lacks such norms due to insufficient knowledge regarding this disease, with the exception of some published case reports.

## 2. Materials and Methods

### 2.1. Samples Collection

A retrospective cross-sectional analytical study was carried out at the National Laboratory of Genomics and Health (LANAGESA) Hidalgo, Mexico, in collaboration with BioGeneticks Laboratory and the “Federico Gomez” Children’s Hospital as well as the Autonomous University of the State of Hidalgo (UAEH-ICAP). Sample collection and selection took place over the period covering September 2017 through October 2019. Blood samples from the radial vein of human subjects were obtained immediately after they signed consent forms, these provided the patients with detailed information about where the blood sample would be drawn from, the amount of blood to be extracted, the assumed tick attachment site, the tests to be conducted, and a confidentiality agreement, which excluded the details of the clinical manifestation, as these would be used for the research being conducted. Patients were selected based on the following criteria: patients manifesting suggestive clinical symptoms, such as fever, headache, nausea, vomiting, abdominal discomfort, diarrhea, arthralgia, myalgia, hepato-splenomegaly, gingivohemorraging, petechial, and increase in transaminase levels, among others, and had no other diagnosis. Patients who presented the necessary clinical symptoms but had other positive diagnoses, as well as those who did not agree to sign consent forms, were eliminated from the test group. Blood samples were obtained using purple tubes (EDTA), separated through centrifugation (5 min at 3500 rpm) for processing. To obtain a sample size representative of the frequency of infection previously reported) [1], of 7% with an α value of 0.05 and confidentiality of 0.95, the software EpinInfo version 7.2.2.6 was used. This requires at least 486 blood samples from patients with suggestive clinical symptoms. All patients with a history of travel abroad were dismissed.

### 2.2. IFA Detection of Anaplasma Phagocytophilum

Samples were analyzed using indirectly acquired immunofluorescence (IFA), to determine the quantity of positive antibodies to immunoglobulins G (IgG) [6]. This was accomplished with the MIF antibody kit of IgG (FULLER Laboratories, Fullerton, CA, USA), following the manufacturer’s indications, with a cut-off point of 1:64. Positive samples were tittered, to the minimum level of positivity, performed using a EUROStar III Plus epifluoromicroscope (EUROIMMUN, Lübeck, Germany).

### 2.3. PCR Detection of Anaplasma Phagocytophilum

The genetic material was extracted by ENZA Blood DNA Mini kit—Buffy coat protocol (OMEGA-BioTek, Norcross, GA, USA). We transferred 250 µL of Buffy coat and 250 µL of 10 mM Tris-HCl and add 25 µL and Ob Protease solution and 500 µL BL buffer, which was vortexed at maximum speed for 15 s and incubated at 65 °C for 10 min and 500 µL 100% Ethanol were added and vortexed 15 s at maximum speed. The DNA column was inserted in a 2 mL collection tube and transferred to a 750 µL sample column; centrifuged at >10,000× *g* for 1 min, the filtrate was discarded and the column was inserted into a new mL collection tube, and 500 µL HBC buffer was added and centrifuged at >10,000× *g* for 1 min, the filtrate was discarded and the collection tube was reused, then 700 µL DNA wash buffer was added and centrifuged at >10,000× *g* for 1 min, the filtrate was discarded and the empty column was centrifuged for 2 min at >10,000× *g* to dry the column matrix. It was transferred into a nuclease-free 2 mL microcentrifuge tube and 200 µL elution buffer was added and heated to 65 °C. It was allowed to sit at room temperature for 5 min and centrifuged at >13,000× *g* for 1 min and stored at −20 °C. All the DNA was evaluated by Nanodrop ONE^c^ (Thermofisher Scientific, Madison, WI, USA), spectrophotometry.

The *A. phagocytophilum* detection was performed using *16SrRNA* and *groEL* genes. Each reaction was set up using 5 μL of DNA, 10 pmol (final per reaction) of each forward (Aph16F 5′-ATAATAGTAGTTGCGTCTTTTGTG-3′) and reverse (Aph16R 5′-ATTCTTTCGCCTTACTTTGTTAC-3′) designed using Primer Select v.11.2.1 program. PCR cycling condition of 1 min, of denaturation at 94 °C, 45 seg of annealing and 1 min of extensión at 72 °C followed by 35 cycles. All the samples that were positive in the *16SrRNA* assay were tested by nested PCR targeting *groEL* gene. The primers used in the nested PCR (HS1a 5′-AITGGGCTGGTAITGAAAT-3′) and reverse (HS6a 5′-CCICCIGGI–CIAIACCTTC-3′) in the first round and primers forward (HS43 5′-AT(A/T)GC(A/T)AA(G/A)GAAGCATAGTC-3′) and reverse (HSVR 5′-CTCAACA–CAGCTCTAGTAGC-3′) amplified a 1297–bp region of the *GroEL* gene, and PCR cycling as reported [10,18]. In both PCR reactions sterile water free of DNase and RNase was used as the negative control and the *A. phagocytophilum* Webster strain (Kindle donated by Donald Bouyer lab) as a positive control in both reactions. PCR amplification analysis was performed using Mastercycler equipment (Eppendorf, Hamburg, Germany). Amplified DNA product was visualized on a 1.5% agarose gel (UltraPure™ Agarose Invitrogen by Life technologies, CA, USA), at 85 volts for 30 min. All positive PCR products for both *16SrRNA* and *GroEL* genes were purified by enzymatic and sequenced in both directions with an ABI BigDye terminator kit (Applied Biosystems, CA, USA) and analyzed on an ABI prism 3130 automated sequencer.

### 2.4. Statistical Analysis

Clinical manifestations were analyzed using a 2 × 2 contingency table with a chi-square value of α 0.05 and confidentiality of 0.95, analyzed using the CDC’s software EpinInfo version 7.2.2.6.

## 3. Results

**Figure 1:**
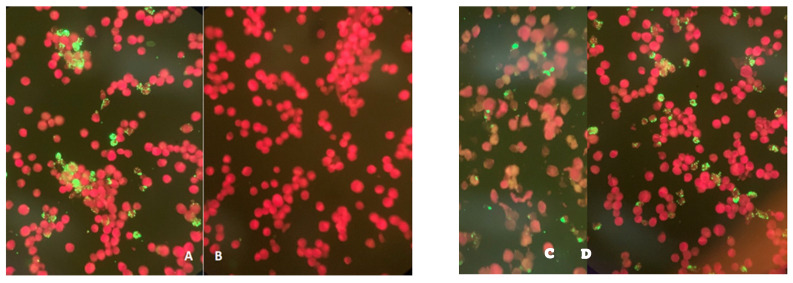
(**A**) and (**B**): Positive and Negative controls. (**C**) and (**D**): positive samples to A. phagocytophilum.

A total of 1924 patients were analyzed; we discarded 910 samples for this study, because the patients reported a history of traveling abroad in a period of 3 weeks before starting clinical manifestations. A total of 1014 blood samples of patients with suggestive clinical manifestations were obtained from 12 states of the Republic of Mexico (State of Mexico, City of Mexico, Sonora, Jalisco, Sinaloa, Hidalgo, Yucatan, Puebla, Veracruz, Michoacan, Morelos, and Chihuahua). Of these, 85 samples (8.38%) were positive for *A. phagocytophilum* per IFA (Figure 1); only 21 (24.71%) positive patients from the initial IFA analysis consented to providing a second sample four weeks after the initial one. A sample of cerebrospinal fluid was taken for the patients with neurological disorder and the results obtained were share for this study.

Serological samples positives from patients by immunofluorescence to *Anaplasma phagocytophilum*.

**Figure 2:**
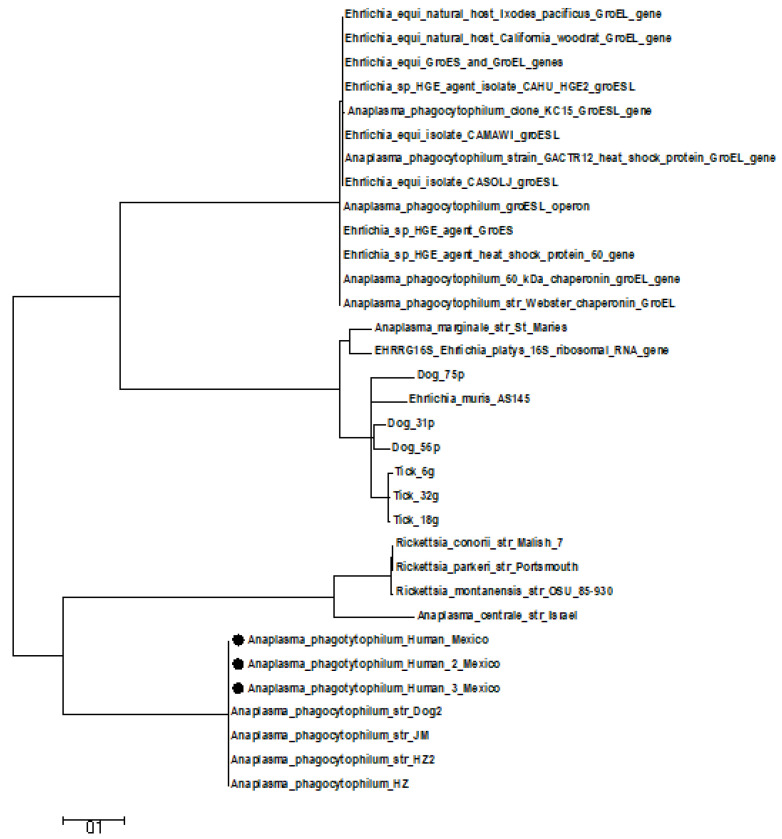
The evolutionary history was inferred by using the Maximum Likelihood method. The tree with the highest log likelihood (−1074.2304) is shown. Initial tree for the heuristic search were obtained automatically by applying Neighbor-Join and BioNJ algorithms to a matrix of pairwise distances estimated using the Maximum Composite Likelihood (MCL) approach, and then selecting the topology with superior log likelihood value. The tree is drawn to scale, with branch lengths measured in the number of substitutions per site. The analysis involved 24 nucleotide sequences. All positions containing gaps and missing data were eliminated. There were a total of 400 positions in the final dataset. Evolutionary analyses were conducted in MEGA5.

Of these, 100% showed increased levels from 1:384 to 1:896. The positive samples for PCR obtained through amplification of the gene *groEL* gene was 7.10% (72 positive samples) and sequenced (MN714906, MN714907 and MN714908; Figure 2).

Phylogenetic analysis of *Anaplasma phagocytophilum* sequences from different specimens by Maximum Likelihood method.

Cerebrospinal fluid of the patients who tested positive, 8, (9.41%) was acquired, and these presented abnormalities as elevated protein levels (81–93 mg/dL), two patients showed evidence of infiltration, glucose was normal and no cells were reported. Significant clinical symptoms included dizziness, vomiting, petechia, epistaxis, enlarged spleen/liver and thrombocytopenia (Table 1). Three percent (3.2%) of the patients required hospitalization of 1.5 days. No fatalities occurred.

## 4. Discussion

In Mexico, the presence of *A. phagocytophilum* in the competent vector, reservoirs of the domestic and wild cycle, such as dogs, mice and opposums, for which it was essential to carry out a search for possible infected patients, as well as the characterization of their clinical manifestations and possible lesions of HGA infection, is why it is important to answer the question of the existence of possible cases, their symptoms, and epidemiological findings. These results further support and provide evidence of the existence of human infection of *Anaplasma phagocytophilum* in Mexico, further confirming previous suggestive serological and molecular tests [1,9,11], these indicated the presence of the bacteria throughout the vector’s environment, as well as reservoirs as dogs and opossums; and accidental human hosts causative of the disease [15,16,17,18,19].

The frequency of infection obtained through the IFA technique is low compared to the United States, where average samples consist of 6.3 cases per million habitants [6,20,21] and Canada [22]. This is consistent, since obligatory records of incidence must be maintained, whereas Mexico has no precedents of this disease with the exception of a case studies [9], and a vector and domestic and wildlife reservoirs reported such a dogs and oposums [21,22,23,24,25]. Employing IFA with antibodies IgG as one of the methods to diagnose the disease may result in low sensitivity for patients in an acute phase. The present study demonstrated the presence of antibodies IgG, since IgM should not be considered as indicative of the infection due to the increased sensibility and decreased specificity of these. A second sample is necessary to demonstrate an elevation of at least four times the antibody titters to be considered true positives [6]; however, in the patients who took the second sample we could notice an increase of three to six times. Evidence exists that the antibodies for *A. phagocytophilum*, may remain elevated up to 4 years after the initial diagnosis. However, only 24.71% of the patients who tested positive consented to a second sampling. All of these resulted in levels 6 to 12 times greater of those obtained from the previous sample. The purpose of this work is to provide evidence of the existence of human patients with HGA, the demonstration of elevated antibodies is fundamental [26]. Of the 85 patients who tested positive, all reported that they had not travelled abroad, this supports that the disease was acquired in Mexico. Only 7.10% of the patients tested positive using PCR. The sensitivity, once the infection is acute, may be low but the specificity of this technique is irrevocable since it demonstrates 100% homology in comparison to other strains already described as being tested for in these patients. It has been shown that *A. phagocytophilum* can circulate in different host tissues and vectors; similar to the fauna found in Mexico. Although human cases have been identified in an ecotype, it is not ruled out that this may increase due to the expansion of the vector [23]. Clinical manifestation is similar to those reported by characteristic patients with the exception of the presence of rashes, subjects from this study reported a 10% incidence of these in comparison to the average, which is lower than 7%. No abnormalities were detected during the sampling of the cerebrospinal fluid, excepting protein levels. No cases of fatality occurred, in comparison with the reported 0.3 fatality in persons over the age of 60. Nevertheless, it is important to note that the average age for patients who tested positive was 52.6 years of age. Despite the fact that patients responded well to the treatment, which consisted of doxycycline, it should be pointed out that ticks transmit other pathogens such as *Babesia spp*. *Borrelia spp*. and *Ehrlichia chaffeensis*, thus co-infections may occur. Cases of *B. burgdorferi* have been reported from the northern part of the country, with some neurological pediatric patients, and one fatality by *E. chaffeensis* in a patient from central Mexico [15,24,26]. In the United States, coinfections are reported in 10% of cases across the country and 6.8% seropositive samples for antibodies against at least one pathogen in Northern California, a western border with Mexico [6,27,28].

It is necessary to conduct further studies that include intentional and systemic testing for potential coinfections in these patients. Efforts to isolate the bacteria were unsuccessful. It is known that *A. phagocytophilum* is a bacterium that is difficult to cultivate in liquid media, as well as the prolonged time required, approximately 1.2 months. Understanding the dynamics of disease transmission in the human–domestic and wildlife cycle in a national serological study is necessary, in which a secondary sample of patients is obtained in conjunction with a risk level map. Nevertheless, this particular study demonstrates sufficient evidence to recognize *A. phagocytophilum* as the causative agent of HGA in Mexico. Health institutions and medical practitioners in general should include diagnostic testing for this infection. In addition, populations at risk should recognize it as an emerging infectious disease transmitted in Mexico.

## Figures and Tables

**Table 1:** Clinical manifestations present in patients with suspicion and positive.

Clinical Manifestations	Patients	HGA (%)	P (IC)
Fever	524	65 (12.40)	1.014 (0.593–1.734)
Rash	219	35 (15.98)	2.309 (1.438–3.706)
Headache	428	61 (14.25)	1.726 (1.046–2.848)
Myalgia	603	38 (06.30)	0.090 (0.054–0.150)
Arthralgia	95	27 (28.42)	26.348 (12.147–57.14)
Asthenia	431	28 (06.50)	0.326 (0.201–0.528)
Dizziness	132	31 (23.48)	6.461 (3.791–11.010)
Nauseas	503	23 (04.57)	0.140 (0.084–0.234)
Vomit	283	51 (18.02)	2.863 (1.795–4.567)
Petechiae	86	11 (12.79)	85.473 (10.878–671.56)
Epistaxis	118	10 (08.47)	2.193 (1.038–4.633)
Spleen/hepatomegaly	216	22 (10.19)	1.186 (0.703–2.001)
Anemia	241	28 (11.62)	1.322 (0.811–2.155)
Uveitis/conjunctivitis	354	46 (12.99)	1.346 (0.852–2.125)
Leucopenia	239	39 (16.32)	2.323 (1.459–3.698)
Plaquetopenia	357	59 (16.53)	2.536 (1.554–4.1379)

% Frequency of clinical manifestation per disease; HGA: Human granulocytic anaplasmosis; P: 0.95 confidence. IC: confidence interval.

## Data Availability

The access number from sequences used to support the findings of this study are included within the article. The serum and DNA used to support the findings of this study are restricted by the ethics committee (UAEH/ICAP/MVyZ-22) in order to protect the Patient privacy. Data are available from Carolina Sosa, PhD., carolina_sosa@uaeh.edu.mx for researchers who meet the criteria for access to confidential data.

## References

[1] Sosa-Gutierrez C.G., Vargas M., Torres J., Gordillo-Perez M.G. (2014). Tick-borne rickettsial pathogens in rodents from Mexico. J. Biomed. Sci. Eng..

[2] Dumler J.S., Choi K.S., Garcia J.C., Barat N.S., Scorpio D.G., Garyu J.W., Bakken J.S. (2005). Human Granulocytic Anaplasmosis and Anaplasma phagocytophilum. Emerg. Infect. Dis..

[3] Nicholson W.L., Allen K.E., McQuiston J.H., Breitschwerdt E.B., Little S.E. (2010). The increasing recognition of rickettsial pathogen in dogs and people. Trends Parasitol..

[4] Guzman N., Beidas S.O. (2019). Anaplasma phagocytophilum (Anaplasmosis). StatPearls.

[5] Bakken J.S., Dumler J.S. (2015). Human granulocytic anaplasmosis. Infect. Dis. Clin. N. Am..

[6] Biggs H.M., Behravesh C.B., Bradley K.K., Dahlgren F.S., Drexler N.A., Dumler J.S., Traeger M.S. (2016). Diagnosis and Management of Tickborne Rickettsial Diseases: Rocky Mountain Spotted Fever and Other Spotted Fever Group Rickettsioses, Ehrlichioses, and Anaplasmosis—United States. MMWR Recomm. Rep..

[7] Graf P.C., Chretien D.L., Ung L., Gaydos J.C., Richards A.L. (2008). Prevalence of seropositivity to Spotted fever group Rickettsiae and Anaplasma phagocytophilum in a agrge, demographically diverse US sample. Clin. Infect. Dis..

[8] Thomas R.J., Dumler J.S., Carlyon J.A. (2009). Current management of human granulocytic anaplasmosis, human monocytic ehrlichiosis and Ehrlichia ewingii Ehrlichiosis. Expert Rev. Anti-Infect..

[9] Sosa-Gutierrez C.G., Cervcantes-Castillo M.A. (2018). First case report of Human Granulocytic Anaplasmosis in Mexico with serological and molecular evidence. Biomed. J. Sci. Tech. Res..

[10] Liz J.S., Sumner J.W., Pfister K., Brossard M. (2002). PCR detection and serological evidence of granulocytic ehrlichial infection in roe deer (*Capreolus capreolus*) and chamois (*Rupicapra rupicapra*). J. Clin. Microbiol..

[11] Sosa-Gutierrez C.G., Vargas-Sandoval M., Torres J., Gordillo-Pérez G. (2016). Tick-borne rickettsial pathogens in questing ticks, removed from humans and animals in Mexico. J. Vet. Sci..

[12] Maggi R.G., Krämer F. (2019). A review on the occurrence of companion vector-borne diseases in pet animals in Latin America. Parasit Vectors.

[13] Álvarez-Hernández G., Roldán J.F.G., Milan N.S.H., Lash R.R., Behravesh C.B., Paddock C.D. (2017). Rocky Mountain spotted fever in Mexico: Past, present, and future. Lancet Infect. Dis..

[14] Álvarez-Hernández G., Candia-Plata M., Delgado-de la Mora J., Acuña-Meléndrez N., Vargas-Ortega A., Licona-Enríquez J. (2016). Fiebre maculosa de las Montañas Rocosas en niños y adolescentes mexicanos: Cuadro clínico y factores de mortalidad. Salud Pública México.

[15] Feria-Arroyo T.P., Castro-Arellano I., Gordillo-Perez G., Cavazos A.L., Vargas-Sandoval M., Grover A., Esteve-Gassent M.D. (2014). Implications of climate change on the distribution of the tick vector Ixodes scapularis and risk for Lyme disease in the Texas-Mexico transboundary region. Parasites Vectors.

[16] Vargas-sandoval M., Priego-Santander A.G., Larrazábal A., Sosa-Gutierrez C.G., Lara-Chavez M.B., Avila-Val T. (2014). Potential sepecies distribution and richness of Ixodidae ticks associated with wild vertebrates from Michoacan, Mexico. J. Geogr. Inf. Syst..

[17] Illoldi-Rangel P., Rivaldi C.L., Sissel B., Trout Fryxell R., Gordillo-Pérez G., Rodríguez-Moreno A., Sarkar S. (2012). Species distribution models and ecological suitability analysis for potential tick vectors of lyme disease in Mexico. J. Trop. Med..

[18] Frans O.L., Wilhelmsson P., Sjöwall J., Jonsson-Henningsson A., Nordberg M., Jørgensen C.S., Krogfelt K.A., Forsberg P., Lindgren P.E. (2020). Emerging tick-borne pathogens in the Nordic countries: A clinical and laboratory follow-up study of high-risk tick-bitten individuals. Ticks Tick-Borne Dis..

[19] Carpi G., Bertolotti L., Pecchioli E., Cagnacci F., Rizzoli A. (2009). *Anaplasma phagocytophilum groEL* gene heterogeneity in *Ixodes ricinus* larvae feeding on roe deer in Northeastern Italy. Vector Borne Zoonotic Dis..

[20] Dewage B.G., Little S., Payton M., Beall M., Braff J., Szlosek D., Knupp A. (2019). Trends in canine seroprevalence to *Borrelia burgdorferi* and *Anaplasma spp*. in the eastern USA, 2010–2017. Parasites Vectors.

[21] Pascoe E.L., Stephenson N., Abigana A., Clifford D., Gabriel M., Wengert G., Brown R., Higley M., Bloch E.M., Foley J.E. (2019). Human Seroprevalence of Tick-Borne Anaplasma phagocytophilum, Borrelia burgdorferi, and Rickettsia Species in Northern California. Vector Borne Zoonotic Dis..

[22] Nelder M.P., Russell C.B., Lindsay R., Dibernardo A., Brandon N.C., Pritchard J., Johnson S., Cronin K., Patel S.N. (2019). Recent Emergence of *Anaplasma phagocytophilum* in Ontario, Canada: Early Serological and Entomological Indicators. Am. J. Trop. Med. Hyg..

[23] Jahfari S., Coipan E.C., Fonville M., van Leeuwen A.D., Hengeveld P., Heylen D., Sprong H. (2014). Circulation of four *Anaplasma phagocytophilum* ecotypes in Europe. Parasites Vector.

[24] Sosa-Gutierrez C.G., Solorzano-Santos F., Walker D.H., Torres J., Serrano C.A., Gordillo-Perez G. (2016). Fatal Monocytic Ehrlichiosis in Woman, Mexico, 2013. Emerg. Infect. Dis..

[25] Gordillo-Perez G., Torres J., Solórzano-Santos F., De Martino S., Lipsker D., Velazquez E., Jaulhac B. (2007). *Borrelia burgdorferi* infection and cutaneous Lyme disease, Mexico. Emerg. Infect. Dis..

[26] Ismail N., Bloch K.C., McBride J.W. (2010). Human ehrlichiosis and anaplasmosis. Clin. Lab. Med..

[27] Rojero-Vazquez E., Gordillo-Perez G., Weber M. (2017). Infection of Anaplasma phagocytophilum and Ehrlichia spp. In Opposums and dogs in Campeche, Mexico: The role of tick infestation. Front. Ecol. Evol.

[28] Movilla R., Garcia C., Siebert S., Roura X. (2016). Countrywide serological evaluation of canine prevalence for *Anaplasma* spp., *Borrelia burgdorferi* (sensu lato), *Dirofilaria immitis* and *Ehrlichia canis* in Mexico. Parasites Vectors.

